# CD90^low^ glioma-associated mesenchymal stromal/stem cells promote temozolomide resistance by activating FOXS1-mediated epithelial-mesenchymal transition in glioma cells

**DOI:** 10.1186/s13287-021-02458-8

**Published:** 2021-07-13

**Authors:** Bing-zhou Xue, Wei Xiang, Qing Zhang, Hao-fei Wang, Yu-jie Zhou, Han Tian, Ahmed Abdelmaksou, Jian Xue, Min-xuan Sun, Dong-ye Yi, Nan-xiang Xiong, Xiao-bing Jiang, Hong-yang Zhao, Peng Fu

**Affiliations:** 1 Department of Neurosurgery, Union Hospital, Tongji Medical College, Huazhong University of Science and Technology, Wuhan, 430022 China; 2 Brain Tumor Research Center, Beijing Neurosurgical Institute, Capital Medical University, Beijing, 100050 China; 3 Department of Neurosurgery, Faculty of Medicine, Helwan University, Cairo, 11435 Egypt; 4 Henan Vocational University of Science and Technology, Zhoukou, 466000 China; 5 Jiangsu Key Lab of Medical Optics, Suzhou Institute of Biomedical Engineering and Technology, Chinese Academy of Sciences, Suzhou, 215163 China

**Keywords:** Glioma, Chemotherapy resistance, EMT, Glioma-associated mesenchymal stromal/stem cells (gaMSCs), FOXS1

## Abstract

**Background:**

The tumour microenvironment contributes to chemotherapy resistance in gliomas, and glioma-associated mesenchymal stromal/stem cells (gaMSCs) are important stromal cell components that play multiple roles in tumour progression. However, whether gaMSCs affect chemotherapy resistance to the first-line agent temozolomide (TMZ) remains unclear. Herein, we explored the effect and mechanism of gaMSCs on resistance to TMZ in glioma cells.

**Methods:**

Human glioma cells (cell line U87MG and primary glioblastoma cell line GBM-1) were cultured in conditioned media of gaMSCs and further treated with TMZ. The proliferation, apoptosis and migration of glioma cells were detected by Cell Counting Kit-8 (CCK-8), flow cytometry and wound-healing assays. The expression of FOXS1 in glioma cells was analysed by gene microarray, PCR and Western blotting. Then, FOXS1 expression in glioma cells was up- and downregulated by lentivirus transfection, and markers of the epithelial-mesenchymal transformation (EMT) process were detected. Tumour-bearing nude mice were established with different glioma cells and treated with TMZ to measure tumour size, survival time and Ki-67 expression. Finally, the expression of IL-6 in gaMSC subpopulations and its effects on FOXS1 expression in glioma cells were also investigated.

**Results:**

Conditioned media of gaMSCs promoted the proliferation, migration and chemotherapy resistance of glioma cells. The increased expression of FOXS1 and activation of the EMT process in glioma cells under gaMSC-conditioned media were detected. The relationship of FOXS1, EMT and chemotherapy resistance in glioma cells was demonstrated through the regulation of FOXS1 expression in vitro and in vivo. Moreover, FOXS1 expression in glioma cells was increased by secretion of IL-6 mainly from the CD90^low^ gaMSC subpopulation.

**Conclusions:**

CD90^low^ gaMSCs could increase FOXS1 expression in glioma cells by IL-6 secretion, thereby activating epithelial-mesenchymal transition and resistance to TMZ in glioma cells. These results indicate a new role of gaMSCs in chemotherapy resistance and provide novel therapeutic targets.

**Supplementary Information:**

The online version contains supplementary material available at 10.1186/s13287-021-02458-8.

## Background

Glioma is the most common highly aggressive and malignant primary brain tumour in adults [1]. Glioblastoma multiforme (GBM), WHO grade IV glioma, has a median overall survival of 14.6 months and a 5-year survival rate of 6.7% after standard treatment of surgery, radiotherapy and chemotherapy [1]. For patients with gliomas, temozolomide (TMZ) is still the first-line chemotherapeutic option [2]. TMZ is an oral alkylating agent that effectively breaks single- and double-stranded DNA, induces cell cycle arrest at the G2/M phase and causes cell apoptosis [2]. Clinical data suggest that the poor prognosis of combined therapy is the result of acquired resistance of glioma tumour cells to TMZ [3]. Several resistance mechanisms, including DNA repair enzyme activity, overexpression of oncogene mutations, involvement of autophagy and the existence of glioma stem cells, have been proven [3–5]. However, the role of the tumour microenvironment in the chemotherapeutic resistance of gliomas is rarely explored [6].

The standard treatment schedule for patients with glioblastoma is TMZ (150 mg/m^2^ on days 1 through 5 and 15 to 19 every 28 days) in the adjuvant phase after surgical resection and radiotherapy [1, 2]. TMZ could cause tumour cell apoptosis through DNA damage [1]. Currently, the prognosis of gliomas is still poor because tumour cells can acquire TMZ resistance [2]. TMZ resistance was largely attributed to DNA repair by MGMT, and MGMT promoter methylation was predicted to be positively associated with better overcomes [2, 4, 7]. However, Yi et al. found that DHC2 (dynein, cytoplasmic 2, heavy chain 1) could repair TMZ-induced DNA damage in MGMT-deficient glioblastomas [8]. Moreover, all glioblastoma patients with methylated and unmethylated MGMT promoters could benefit from TMZ treatment [2, 9]. Therefore, the mechanism of TMZ resistance remains unclear, especially the contribution of the tumour microenvironment to TMZ resistance in gliomas [4, 10, 11].

In human gliomas, nonneoplastic cells can be recruited into the stromal environment by neoplastic tumour cells from the normal tissues and bone marrow, and these stromal cells contribute to several hallmark capabilities, such as sustaining proliferative signalling, resisting cell death, inducing angiogenesis, activating invasion and metastasis, evading immune destruction and acquiring therapeutic resistance [6, 12]. Mesenchymal stromal/stem cells (MSCs) were proven to migrate towards glioma cells under the guidance of angiogenic cytokines in vivo and in vitro [13]. When MSCs are recruited into the tumour microenvironment, they can be induced into glioma-associated MSCs (gaMSCs) by cellular crosstalk to contribute to tumour progression in gliomas [13]. Tal et al. found that a high percentage of gaMSCs in newly diagnosed high-grade glioma patients correlated with worse overall survival [14]. Anwar et al. reported that gaMSCs could drive the proliferation of gliomas and maintain the stemness of glioma stem cells by secreting IL-6 and activating STAT3 [15]. Javier et al. reported that gaMSCs could increase the tumourigenicity of glioma stem cells through miR-1587-containing exosomes [16]. Our group also found that two subpopulations, CD90^low^ gaMSCs and CD90^high^ gaMSCs, played different roles in tumour progression [17–19]. On the other hand, MSCs were proven to contribute to therapy resistance in a wide variety of tumours [20, 21]. However, whether gaMSCs contribute to chemotherapeutic resistance to TMZ remains unclear.

Herein, we first demonstrate that the increased expression of FOXS1 is positively correlated with the effect of TMZ on glioma cells cultured in conditioned media (CM) of gaMSCs. Moreover, the increased expression of FOXS1 was sufficient to activate epithelial-mesenchymal transition and induce TMZ resistance in vivo and in vitro. We then show that FOXS1 expression in glioma cells was increased by secretion of IL-6 in conditioned media, which originated mainly from the CD90^low^ gaMSC subpopulation. The current findings suggest that CD90^low^ glioma-associated mesenchymal stromal/stem cells promote chemotherapeutic resistance by activating FOXS1-mediated epithelial-mesenchymal transition in glioma cells.

## Materials and methods

### Glioma cells (glioma cell line U87MG and human primary glioblastoma cells GBM-1) and gaMSCs

The study was approved by the local ethics committee of Tongji Medical College, Huazhong University of Science and Technology. Written informed consent was obtained from each patient. As we described previously [17], human primary glioblastoma cells and gaMSCs were isolated and cultured. In brief, for gaMSCs, fresh glioma specimens were washed with phosphate-buffered saline (PBS, HyClone, USA) and then processed for mechanical cutting and trypsin (BIYUNTIAN, China) digestion. The mononuclear cells were collected by Ficoll (2:1 Genview, USA) density gradient centrifugation and cultured in DMEM (HyClone, USA) containing 20% foetal bovine serum (FBS, BI, Israel) and 1% penicillin and streptomycin in a humidified atmosphere at 37 °C containing 5% CO2. For primary glioblastoma cells, fresh glioma specimens were processed for washing and digestion. Then, the obtained cell suspension was lysed with erythrocyte lysate (BIYUNTIAN, China), seeded into a neuroblast medium (containing N2, B27, hEGF, FGF and heparin) and transferred to an extracellular matrix culture flask (Corning, USA). The cells were cultured in a humidified atmosphere at 37 °C containing 5% CO2. U87MG cells were purchased from the American Type Culture Collection (ATCC, Gaithersburg, MD, USA) and cultured in DMEM containing 10% foetal bovine serum.

### Conditioned medium collection and magnetic-activated cell sorting (MACS) of gaMSCs

To sort the subpopulations, gaMSCs were collected by Accutase (StemCell, Canada) digestion and incubated with CD90 magnetic bead antibody (Miltenyi, Germany) for 15 min at 4 °C. CD90^high^ gaMSCs and CD90^low^ gaMSCs were obtained by magnetic separation with the autoMACS Pro Separator (Miltenyi, Germany) as previously described [17]. To collect the CM, gaMSCs at a 50% density were washed with PBS 2–3 times and cultured in serum-free DMEM for 3 days. Then, cell supernatants were collected after centrifugation to remove the debris and stored at −20 °C for further experiments.

### Proliferation, migration and apoptosis assay of glioma cells

The proliferation ability was analysed using the Cell Counting Kit-8 (CCK-8 Kit, Dojindo Laboratories, Japan) assay. Glioma cells (5000 cells/well) were seeded into 96-well plates (Corning, USA) at 100 μl and incubated overnight. Then, the medium was changed to serum-free DMEM medium, and TMZ was added as scheduled. Ten microlitres of CCK-8 solution was added at various time points, and the absorbance value at 450-nm wavelength was detected by a microplate reader (PerkinElmer, USA) after 2 h of incubation. The migration ability was investigated using a wound-healing assay. Glioma cells were seeded into 6-well plates and incubated until they reached 90–100% confluence. A 10-μl pipette tip was used to carefully make cross lines, and the debris was washed away with PBS. Different medium with 200 μM TMZ was added. The areas of the scratch wounds were imaged with an Olympus microscope at 0 and 24 h and analysed using ImageJ software (NIH, USA). The apoptosis ability was analysed by flow cytometry (FCM). Glioma cells were treated with TMZ for various times and then collected. Glioma cells were washed and processed to FCM by using an annexin V-APC/7-AAD or annexin V-FITC/PI apoptosis assay kit (Nanjing KeyGEN, China) in accordance with the manufacturer’s instructions.

### qRT-PCR

Total RNA was extracted using TRIzol (Ambion, USA). cDNA synthesis and real-time PCR were performed using the SYBR® Premix Ex Taq™ Kit (Takara, Japan). The reaction conditions of reverse transcription were as follows: 25 °C for 5 min, 50 °C for 15 min, 85 °C for 5 min and 4 °C for 10 min. The PCR conditions were as follows: 50 °C for 2 min, 95 °C for 10 min and 40 cycles of 95 °C for 30 s and 60 °C for 30 s. The dissolution curve was generated, and the final data were analysed with 2^−ΔΔCt^. β-Actin was used as an internal control. The sequence of primers for FOXS1 was as follows: forward, 5′-CCCAGGGTTCCTTGTGGTC-3′; reverse, 5′-CCCAGGGTTCCTTGTGGTC-3′. Three independent RNA samples were used for qRT-PCR in triplicate.

### Western blot assay

The proteins of cells or tissue were extracted with RIPA lysis buffer (BIYUNTIAN, China). The protein concentration was measured using a BCA protein concentration assay kit (BIYUNTIAN, China) according to the instructions. Protein electrophoresis was carried out in 5–12% gradient gels. Then, the proteins were transferred onto a PVDF membrane. After membrane transfer, antibodies against FOXS1 (1:1000, Thermo, USA), E-cadherin (1:1000, Abcam, China) and N-cadherin (1:1000, Abcam, China) were incubated overnight at 4 °C. The HRP-labelled secondary antibody (1:50000, BOSTER, China) was then incubated for 2 h at room temperature. Protein bands were detected with an X-ray film and analysed.

### Transfection of cells

Lentiviruses with knockdown or overexpression of FOXS1 and negative control lentiviruses (Shanghai GeneChem Co., LTD.) were manufactured according to the instructions. In brief, cell suspensions (50,000 cells/ml, 2 ml/well) of glioma cells were inoculated into 6-well plates for 24 h in DMEM with 10% FBS. Then, DMEM without FBS was replaced, lentivirus solution (20 μl 1 × 10^8^ TU/ml) was added for another 16-h incubation and DMEM with 10% FBS was added back for another 72 h. The screening and purification of infected cells was performed by culture in a medium containing puromycin.

### Immunohistochemistry

The tumour tissue specimens were fixed in 4% paraformaldehyde and embedded in paraffin after collection from sacrificed mice. Sections were incubated overnight at 4 °C with anti-Ki67 antibody (1:100, Proteintech, China) after dewaxing and antigen retrieval. After incubation with the HRP-conjugated secondary antibody (1:1, Boster, China), the sections were processed for chromogenic development with DAB chromogenic solution (Boster, China). Then, the sections were counterstained using haematoxylin and imaged with an Olympus microscope.

### ELISA

IL-6 levels were measured in different media of gaMSCs, CD90^high^ gaMSCs and CD90^low^ gaMSCs using an ELISA kit (Neobioscience, China) as described in the manufacturer’s instructions. The absorbance was measured at 450 nm. Three independent medium samples were used for ELISA performed in triplicate.

### RNA extraction and Clariom D microarray

Total RNA from U87MG cells was extracted using TRIzol (Invitrogen, USA). U87MG glioma cells were cultured in DMEM without FBS and in conditioned media of gaMSCs for 3 days, and each group had three samples in the same culture conditions. Gn-GeneChip Human Clariom D Array 2.0 (Affymetrix, USA) was used for GeneChip analysis. An Agilent RNA 6000 Nano Kit was used for RNA quality inspection. The GeneChip WT PLUS Reagent Kit was used for the experiment, and the GeneChip Hybridization Wash and Stain Kit was used for chip hybridization and staining. The microarray data were measured and summarized using Clariom D QC tool software (Affymetrix, USA).

### Tumour-bearing mouse experiments

All mouse experimental procedures were conducted following institutional guidelines under approved protocols. Four-week-old male BALB/c-nu mice were purchased from Beijing Vital River Laboratory Animal Center and raised in the animal facilities of Huazhong University of Science and Technology under suitable conditions. For tumour-bearing mouse experiments, mice were anaesthetized by intraperitoneal injections of chloral hydrate (2.5 ml/kg) for intracranial implantation of glioma cells. A total of 5 × 10^5^ glioma cells were injected into the right frontal lobe (2 mm lateral and 1 mm anterior to the bregma, 3.5 mm depth from the skull base) of the mice using a Hamilton syringe (Hamilton Company, USA). After 3 weeks, mice were treated with TMZ (50 mg/kg) by intraperitoneal injection for 5 consecutive days. Mice were sacrificed 35 days later, and the tumour was removed for further experiments.

### Statistical analysis

In the present study, GraphPad Prism software version 8.0 was used for all statistical analyses. All assays were performed in triplicate at least three times unless specifically noted, and the values are presented as the means ± SEM in all tests. The error bars represent the SDs in the figures. The statistical tests involving two experimental conditions were assessed by the unpaired Student’s t test (two-sided), whereas tests involving three or more experimental conditions were assessed by one-way ANOVA followed by Tukey’s test. The survival curve was compared with the log-rank test. P < 0.05 was considered statistically significant.

## Results

gaMSCs were successfully isolated from fresh tissues of patients with different grade gliomas and identified by classical MSC characteristics (these cells express CD105, CD73 and CD90 and differentiate to osteoblasts, adipocytes and chondroblasts in vitro), as described in our previous reports (data not shown) [17, 18].

### Glioma cells cultured in gaMSC-conditioned media show increased TMZ resistance ability

**Fig. 1:**
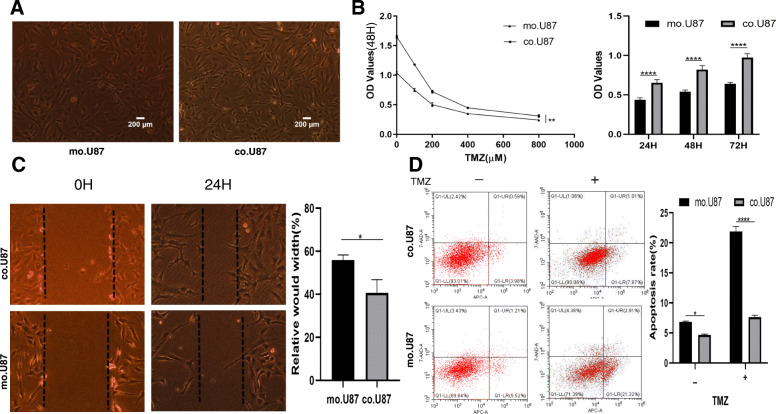
Glioma cell line U87MG show increased TMZ resistance ability in gaMSC-conditioned media in vitro. **A** Morphology of U87MG in normal condition (mo.U87) and gaMSC-conditioned media (co.U87) (× 40, scale bar = 200 μm). **B** Proliferation curve of mo.U87 and co.U87 with different concentrations of TMZ (n ≥ 3, **P < 0.01, ****P < 0.0001). **C** Wound-healing assay of mo.U87 and co.U87 cells under TMZ treatment. n ≥ 3, *P < 0.05. **D** Apoptosis of mo.U87 and co.U87 cells under TMZ treatment. - no TMZ, + add TMZ (n ≥ 3, *P < 0.05, ****P < 0.0001)

To investigate the effect of gaMSCs on the tumour behaviour of glioma cells, the media of gaMSCs isolated from glioblastoma specimens were collected for glioma cell (cell line U87MG and primary glioblastoma cell line GBM-1) culture in this study. As shown in Fig. 1A, compared with that of glioma cells cultured under normal conditions (mo.U87), the cell morphology of U87MG cells was not different after 3 days of indirect coculture with gaMSCs (co.U87). However, we found that their resistance to TMZ was significantly different and that the proliferation capacity of co.U87 cells was stronger than that of mo.U87 cells treated with different concentrations of TMZ (from 200 to 800 μM). co.U87 cells showed increased proliferation ability under the condition of 200 μM TMZ for different times (from 24 to 72 h) in vitro (Fig. 1B). Furthermore, wound-healing assays and flow cytometry were performed on glioma cells treated with 200 μM TMZ, and the results showed that co.U87 cells had significantly increased migration ability (Fig. 1C, D). In addition, the apoptosis rate of co.U87 cells was lower than that of mo.U87 cells with or without TMZ (Fig. 1D). Similarly, primary glioblastoma cells showed increased proliferation and resistance to apoptosis after culture in conditioned media of gaMSCs, as shown in Suppl. Fig. 1. These data suggest that glioma cells acquire increased chemotherapeutic resistance ability in gaMSC-conditioned media.

### Indirect coculture with gaMSCs results in FOXS1 overexpression in glioma cells

**Fig. 2:**
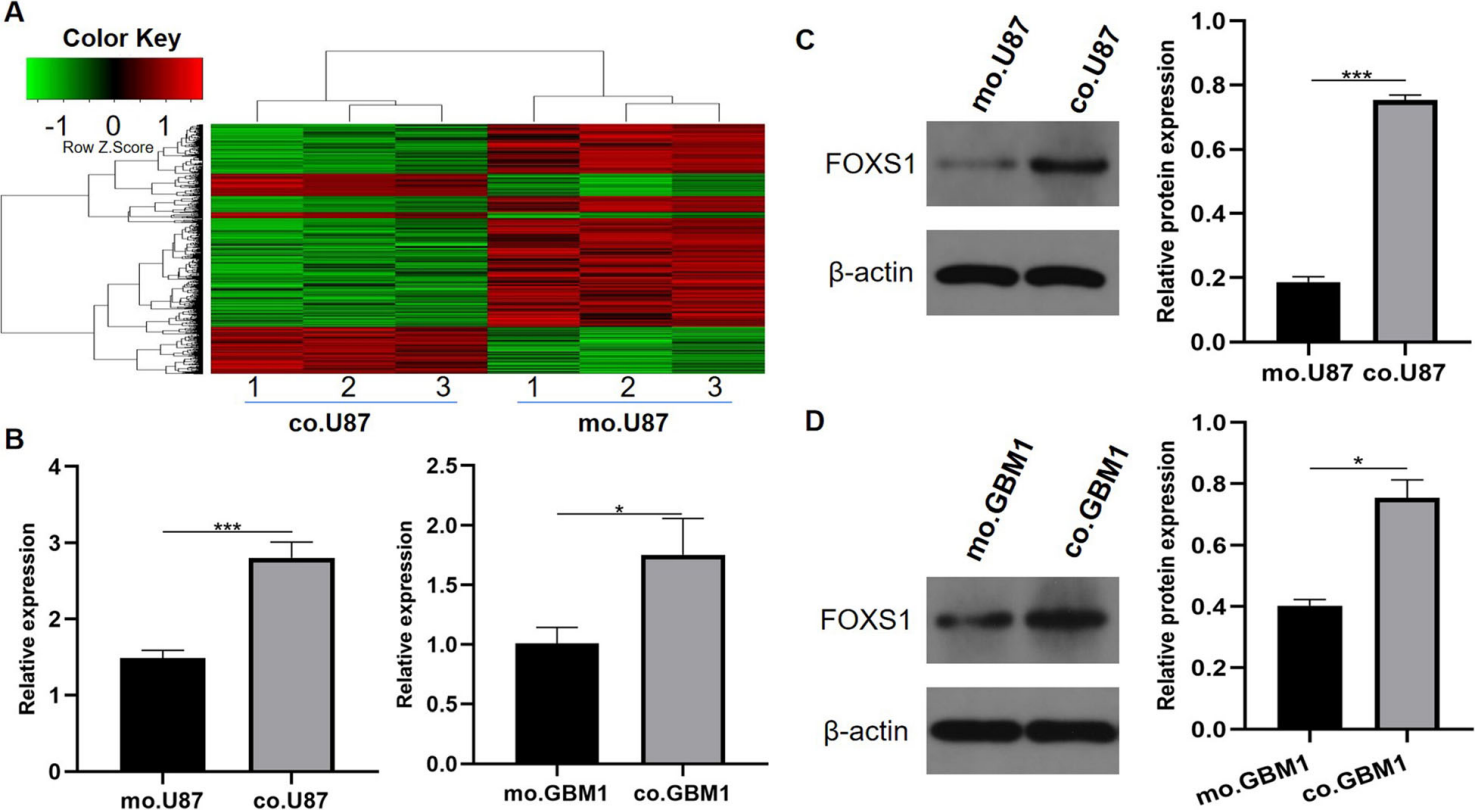
Increased FOXS1 expression in glioma cells after cultivation in gaMSC-conditioned media. **A** Heat map of gene expression differences between mo.U87 and co.U87. The cells were cultured in DMEM (mo.U87) and gaMSC-conditioned media (co.U87) for 3 days and then detected and statistically analysed by microarray. “Red” indicates high relative expression, and “green” indicates low relative expression (*n* = 3). **B** PCR was used to detect FOXS1 expression in glioma cells under different conditions (n ≥ 3, *P < 0.05, ***P < 0.001). **C** Western blot was used to detect FOXS1 expression in U87MG under different conditions (n ≥ 3, ***P < 0.001). **D** Western blot was used to detect FOXS1 expression in GBM-1 under different conditions (n ≥ 3, *P < 0.05)

To determine the underlying molecular mechanism of increased chemotherapeutic resistance of glioma cells after culture in conditioned media of gaMSCs, the changes in gene expression between the mo.U87 and co.U87 groups were investigated by gene microarray (Fig. 2A). The results identified a total of 1251 genes (486 upregulated and 765 downregulated in the co.U87 group). Among them, CA9, FOXS1 and OLFML3 were the top three upregulated genes, and SERPINB4, DTL and PDE1A were the top three downregulated genes. MGMT promoter methylation is the most predictive factor associated with longer survival in patients with glioblastoma who receive TMZ, and the gene microarray showed that the expression of MGMT was not changed after indirect coculture with gaMSCs (P > 0.05). Furthermore, we confirmed the variation in FOXS1 expression at the mRNA and protein levels in glioma cells (both in the U87MG cell line and in GBM-1 primary glioblastoma cells) when glioma cells were cultured in conditioned media of gaMSCs for 3 days (Fig. 2B–D).

### FOXS1 overexpression induces TMZ resistance in glioma cells

**Fig. 3:**
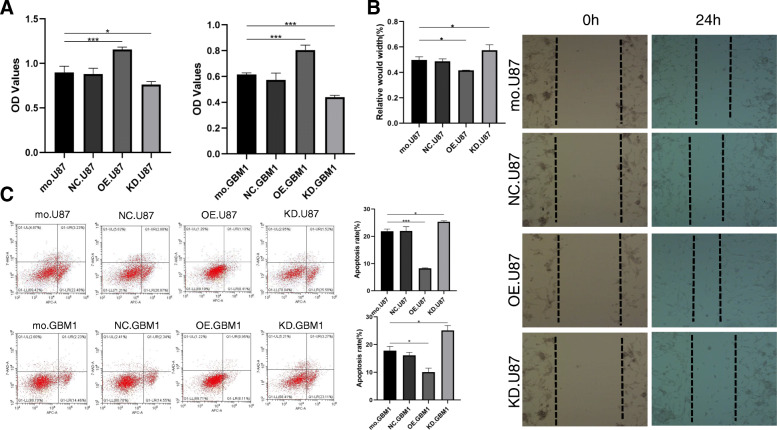
FOXS1 overexpression induces TMZ resistance in glioma cells. The proliferation (**A**), wound-healing (**B**) and apoptosis assay (**C**) of glioma cells with different FOXS1 expression under 200 μM TMZ treatment. control, normal control; NC, negative control; KD, knockdown; OE, overexpression (n ≥ 3, *P < 0.05, ***P < 0.001)

To determine whether FOXS1 overexpression directly induced TMZ resistance in glioma cells, we increased and reduced FOXS1 expression in glioma cells (both in the U87MG cell line and in GBM-1 primary glioblastoma cells) by lentivirus infection, and four glioma cell groups with different FOXS1 expression were obtained (control, negative control/NC, overexpression/OE and knockdown/KD) for further study. We showed that the proliferation, migration and apoptosis resistance ability of U87 and GBM-1 cells treated with 200 μM TMZ were positively correlated with the overexpression of FOXS1, as shown in Fig. 3. On the other hand, low expression of FOXS1 in glioma cells induced increased sensitivity to TMZ. Overall, we found that gaMSCs could increase the TMZ resistance of glioma cells via overexpression of FOXS1.

### FOXS1 overexpression mediates EMT activation in glioma cells

**Fig. 4:**
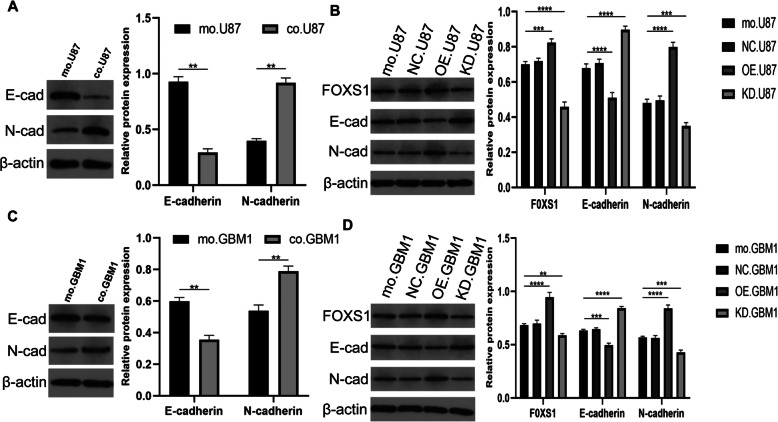
FOXS1 overexpression mediates EMT process activation in glioma cells. Glioma cells cultivated in gaMSC-conditioned media with FOXS1 expression were measured of EMT marker expression by western blot (**A**, **C**). The EMT markers are changed according to FOXS1 expression regulated by lentivirus infection (**B**, **D**) (n ≥ 3, **P < 0.01, ***P < 0.001, ****P < 0.0001)

EMT is a key hallmark in tumour progression that contributes to chemotherapeutic resistance [22]. To determine whether the EMT process participated in the inducing effect of FOXS1 overexpression on TMZ resistance in glioma cells, EMT markers (E-cadherin and N-cadherin) were detected by western blot in glioma cells treated with TMZ. First, we compared the levels of EMT markers in glioma cells in monoculture and indirect coculture with gaMSCs, and the results confirmed the upregulation of N-cadherin and downregulation of E-cadherin and the activation of the EMT process, as shown in Fig. 4A and C. Second, we investigated the changes in EMT markers when the expression of FOXS1 was changed by lentivirus infection. The results showed that the EMT process was activated in glioma cells with FOXS1 overexpression (OE-U87 and OE-GBM1) and suppressed in glioma cells with FOXS1 low expression (KD-U87 and KD-GBM1) when the cells were treated with TMZ. There was no significant difference in the negative control group (NC-U87 and NC-GBM1) (Fig. 4B, D).

### FOXS1 overexpression activates the EMT process and induces TMZ resistance in tumour-bearing mice

**Fig. 5:**
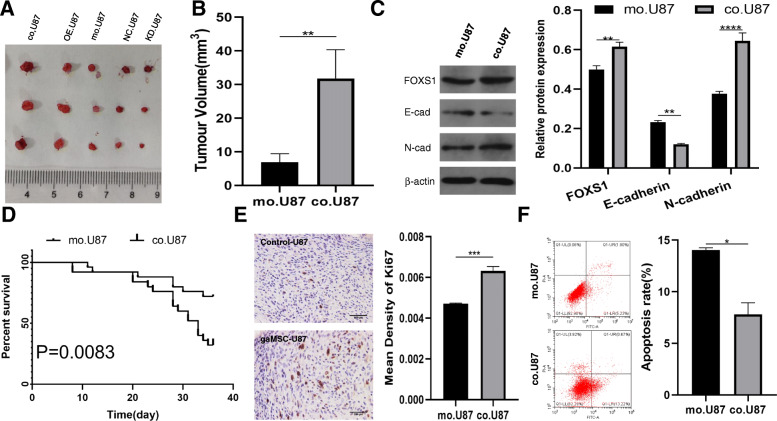
Glioma cells cultivated in gaMSC-conditioned media show activated EMT process and increased TMZ resistance ability in tumour-bearing mice. Representative mice from intracranial xenograft experiments in which U87 cells at different conditions were injected into the right frontal lobes of nude mice, tumour sizes of U87MG treated with TMZ were showed in **A** (*n* = 3). Then, tumour volume (**B**), EMT markers (**C**), survival time (**D**), Ki67-staining (**E**) and apoptosis assay (**F**) were investigated in tumour-bearing mice inoculated with mo.U87/co.U87 and treated with TMZ (× 400, scale bar = 250 μm, *n* = 3, *P < 0.05, **P < 0.01, ***P < 0.001, ****P < 0.0001)

To investigate the relationship of FOXS1 expression, EMT process activation and TMZ resistance in vivo, tumour-bearing mice were generated using male BALB/c-nu mice and the glioma cell line U87MG under various pre-treatment conditions. Then, these tumour-bearing mice were treated by injection with TMZ (50 mg/kg) for 5 consecutive days, and the effect was measured by tumour size, survival time, Ki-67 staining and apoptosis assay. Compared with co.U87 cells, which were cultured in gaMSC-conditioned media, U87 cells cultured under normal conditions had a smaller tumour volume, lower expression of FOXS1, lower expression of the proliferative index Ki-67 and lower apoptosis resistance ability and were associated with longer survival time (Fig. 5A, B, D–F). Moreover, the upregulation of N-cadherin and downregulation of E-cadherin in co.U87MG cells proved the activation of the EMT process during TMZ resistance (Fig. 5C).

**Fig. 6:**
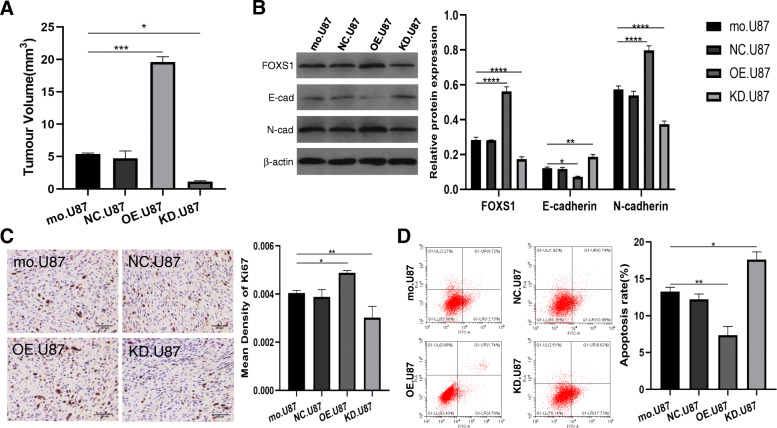
Glioma cells with different FOXS1 expression show activated EMT process and increased TMZ resistance ability in tumour-bearing mice. Tumour volume (**A**), EMT markers (**B**), Ki67-staining (**C**) and apoptosis assay (**D**) were investigated in tumour-bearing mice inoculated with control-U87/NC-U87/KD-U87/OE-U87 and treated with TMZ (× 400, scale bar = 250 μm, *n* = 3, *P < 0.05, **P < 0.01, ***P < 0.001, ****P < 0.0001)

Similar results were observed in tumour-bearing mice established using lentivirus-infected U87MG cells. The Ki-67 staining, tumour volume, apoptosis assay and EMT marker expression of tumour-bearing mice in different FOXS1 expression groups also confirmed that FOXS1 mediated EMT-induced TMZ resistance in vivo, as shown in Fig. 6.

### FOXS1 overexpression in glioma cells is induced by IL-6 mainly secreted by CD90^low^ gaMSCs

**Fig. 7:**
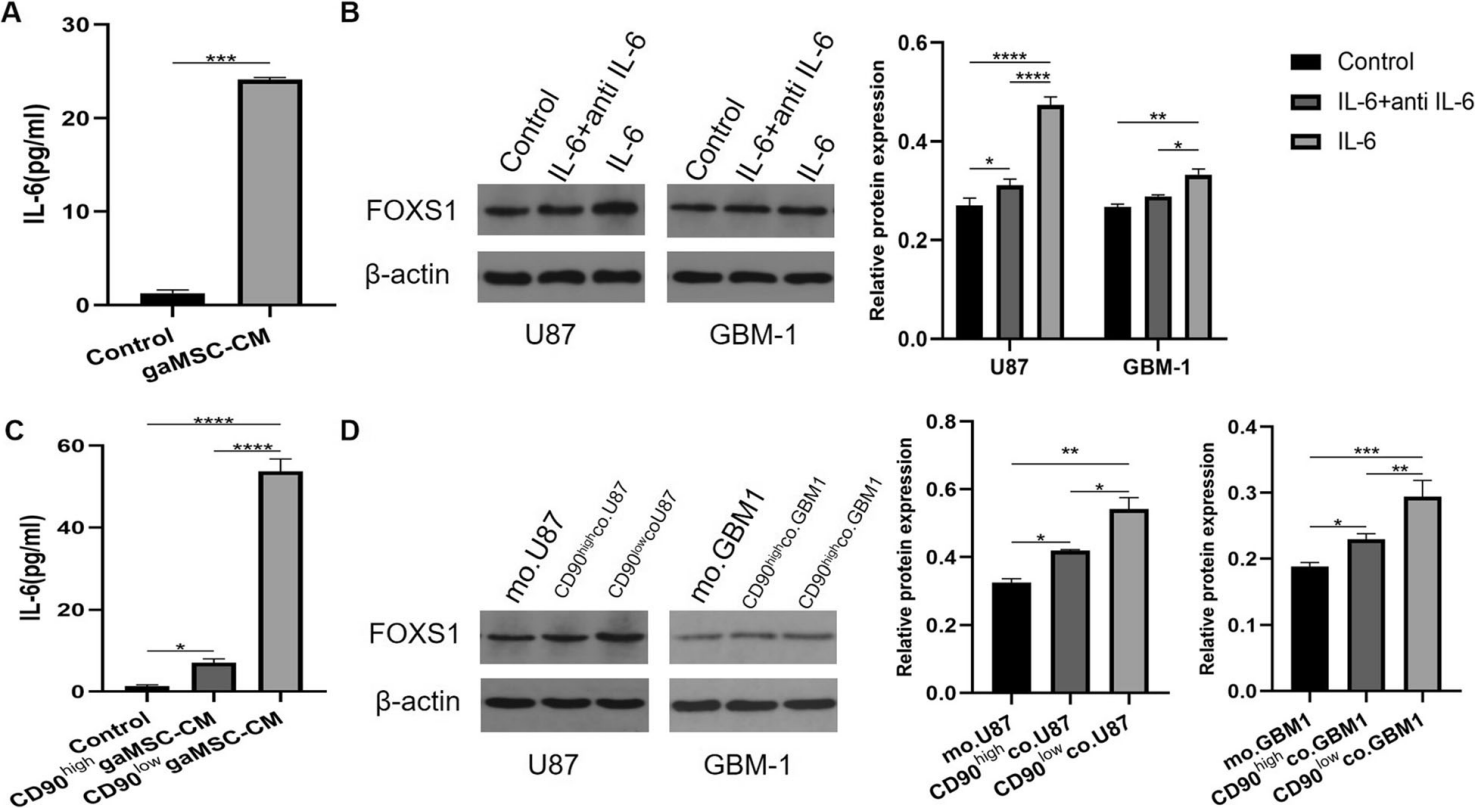
IL-6 which increased FOXS1 expression in glioma cells is mainly secreted by CD90^low^ gaMSCs. IL-6 secretion in conditioned media of gaMSCs, CD90^low^gaMSCs and CD90^high^gaMSCs was measured by ELISA (**A**, **C**). The effect of IL-6 on FOXS1 expression in glioma cells could be reversed by IL-6 antibody (**B**). The FOXS1 expression in glioma cells is higher in conditioned media of CD90^low^gaMSCs than that of CD90^high^gaMSCs (**D**). (n ≥ 3, *P < 0.05, **P < 0.01, ***P < 0.001, ****P < 0.0001)

Anwar et al. reported that IL-6 was the dominant growth factor in the conditioned medium of gaMSCs to promote glioma stem cell proliferation and self-renewal [15]. We confirmed that a high concentration of IL-6 could be secreted by gaMSCs in the present study (Fig. 7A). To determine whether IL-6 is the factor by which gaMSCs induce the increased expression of FOXS1 in glioma cells, U87MG and GBM-1 glioma cells were pretreated with recombinant IL-6 with/without IL-6 neutralizing antibody (5 μg/ml) in DMEM and then the expression of FOXS1 was measured. IL-6 increased the expression of FOXS1 in glioma cells compared to the control (DMEM medium) group, similar to gaMSCs. The effect could be neutralized by the IL-6 antibody (Fig. 7B).

Our group previously described the different roles of gaMSC subpopulations, CD90^high^ gaMSCs and CD90^low^ gaMSCs, in tumour progression [17]. We measured the concentration of IL-6 in conditioned media of the CD90^high^ gaMSC and CD90^low^ gaMSC subpopulations and found that the CD90^low^ gaMSC subpopulation secreted a higher level of IL-6 than CD90^high^ gaMSCs (Fig. 7C). We subsequently compared the expression of FOXS1 in glioma cells that were cultured indirectly with the CD90^high^ gaMSC and CD90^low^ gaMSC subpopulations. The results showed that the CD90^low^ gaMSC subpopulation had a higher capacity to increase FOXS1 expression in glioma cells, both U87 and GBM-1 (Fig. 7D). These findings demonstrate that FOXS1 overexpression in glioma cells is induced by IL-6, which is mainly secreted by the CD90^low^ gaMSC subpopulation.

## Discussion

In the present study, we demonstrate a novel mechanism that does not depend on MGMT expression in gliomas. The CD90^low^ gaMSC subpopulation, which is a newly identified recruited component in the tumour microenvironment, secreted IL-6, which then upregulated FOXS1 expression and activated the EMT process, thus contributing to TMZ resistance in glioma cells.

MSCs have been shown to be potent mediators of chemotherapeutic resistance in several cancers [12, 20, 21, 23]. Jeanine et al*.* found that MSCs induced resistance to chemotherapy through fatty acid release in colon carcinoma cells and Lewis lung carcinoma cells [21]. Saradhi et al*.* reported that MSCs exposed to tyrosine kinase inhibitors acquired chemotherapeutic resistance with the expression of genes encoding chemoattractants, adhesion molecules and prosurvival growth factors in acute lymphoblastic leukaemia [23]. gaMSCs were identified to have classical MSC characteristics and were shown to promote tumour progression by multiple cellular crosstalk with neoplastic cells in the glioma microenvironment [19]. Among them, CD90^high^ gaMSCs could increase the proliferation, migration and adhesion of glioma cells, while CD90^low^ gaMSCs could differentiate into pericytes and stimulate the vascular formation of endothelial cells [17, 18]. The present study demonstrated the first evidence that gaMSCs, especially the CD90^low^ gaMSC subpopulation, are involved in the therapy response of human gliomas. This finding is in line with the clinical conclusion that a higher percentage of gaMSCs predicts poorer glioma patient prognosis [14]. Based on this finding, the clinical use of MSC-based therapy is still in its infancy [24].

In the current study, the gene microarray results showed that MGMT expression was not changed when glioma cells were cultured in gaMSC-conditioned media, so TMZ resistance was not related to MGMT expression and needs to be better understood. FOXS1 is a novel oncogene in the regulatory network of tumour progression [25, 26]. Here, we detected increased expression of FOXS1 in glioma cells, both the U87MG cell line and GBM-1 primary glioblastoma cell line, after indirect coculture with gaMSCs at the gene and protein levels. Furthermore, we proved that FOXS1 upregulation was related to TMZ resistance in glioma cells. Previous reports described that EMT is a key process involved in chemotherapeutic resistance [22], and a close correlation between FOXS1 and the EMT process in tumour progression has been reported [25]; therefore, we investigated the underlying mechanism by which FOXS1 activates the EMT process. We upregulated and downregulated the expression of FOXS1 by lentivirus transfection. The markers of the EMT process were changed in response, and the results suggested that FOXS1 activated the EMT process, thus contributing to TMZ resistance in glioma cells. Furthermore, we generated tumour-bearing mice using glioma cells cultured under various conditions. Glioma cells cultured with gaMSC-conditioned media were associated with lower survival, larger tumours, higher Ki67 expression and increased EMT markers than the control group. Glioma cells with high FOXS1 expression formed larger tumours, expressed more Ki67 and were associated with shorter survival and EMT process activation, while glioma cells with low FOXS1 expression showed the opposite effects. Therefore, these findings demonstrate that gaMSCs could increase FOXS1 expression and activate the EMT process, thus conferring TMZ resistance to glioma cells.

gaMSCs secrete several cytokines and exosomes into the tumour microenvironment, such as IL-6, CCL-5 and VEGFA [15–17]. Moreover, the two subpopulations of gaMSCs had completely different expression patterns of secreted factors and related lncRNAs that might affect their roles in glioma progression [15]. IL-6 is a soluble glycosylated polypeptide chain and one of the major components of the tumour microenvironment that can be secreted by different neoplastic cells and stromal cells [15]. In our study, we found that IL-6 in gaMSCs could increase the expression of FOXS1 in glioma cells, while the addition of an IL-6 neutralizing antibody could reverse this effect. Furthermore, higher expression of IL-6 was detected in CD90^low^ gaMSCs than CD90^high^ gaMSCs. After indirect culture with CD90^low^ gaMSCs, glioma cells had higher expression of FOXS1 and EMT markers. Our data suggest that IL-6, mainly originating from CD90^low^ gaMSCs, could increase the expression of FOXS1 in glioma cells and induce TMZ resistance. However, whether IL-6 is the principal factor in the underlying mechanism is still unknown, and the source of IL-6 remains controversial.

## Conclusions

In conclusion, our study reveals a previously undocumented mechanism by which CD90^low^ gaMSCs in the tumour microenvironment contribute to TMZ resistance without dependency on MGMT expression. gaMSCs can increase FOXS1 expression and thus activate the EMT process in gliomas. The stimulation was due to IL-6, which is mainly secreted by CD90^low^ gaMSCs. These findings suggest that combined TMZ and FOXS1 or gaMSC inhibitory treatment could provide a new and effective therapy for reducing or reversing the chemotherapy resistance of gliomas.

## Supplementary Information

**Additional file 1: Supplementary Figure 1.** Glioblastoma primary cell GBM-1 show increased TMZ resistance ability in gaMSCs-conditioned media in vitro. Proliferation curve (A) and apoptosis assay (B) of mo.GBM-1 and co. GBM-1 under TMZ treatment .(n ≥ 3,**P < 0.01,***P < 0.001,****P < 0.0001)

## Data Availability

All data generated or analysed during this study are included in this published article [and its supplementary information files].

## Abbreviations

gaMSCs: Glioma-associated mesenchymal stromal/stem cells
TMZ: Temozolomide
GBM: Glioblastoma multiforme
PBS: Phosphate-buffered saline
FBS: Foetal bovine serum
EMT: Epithelial-mesenchymal transformation
