# Correction to: CD90^low^ glioma-associated mesenchymal stromal/stem cells promote temozolomide resistance by activating FOXS1-mediated epithelial-mesenchymal transition in glioma cells

**DOI:** 10.1186/s13287-021-02566-5

**Published:** 2021-09-06

**Authors:** Bing-zhou Xue, Wei Xiang, Qing Zhang, Hao-fei Wang, Yu-jie Zhou, Han Tian, Ahmed Abdelmaksou, Jian Xue, Min-xuan Sun, Dong-ye Yi, Nan-xiang Xiong, Xiao-bing Jiang, Hong-yang Zhao, Peng Fu

**Affiliations:** 1 Department of Neurosurgery, Union Hospital, Tongji Medical College, Huazhong University of Science and Technology, Wuhan, 430022 China; 2 Brain Tumor Research Center, Beijing Neurosurgical Institute, Capital Medical University, Beijing, 100050 China; 3 Department of Neurosurgery, Faculty of Medicine, Helwan University, Cairo, 11435 Egypt; 4 Henan Vocational University of Science and Technology, Zhoukou, 466000 China; 5 Jiangsu Key Lab of Medical Optics, Suzhou Institute of Biomedical Engineering and Technology, Chinese Academy of Sciences, Suzhou, 215163 China

## Correction to: Stem Cell Research & Therapy (2021) 12:394 https://doi.org/10.1186/s13287-021-02458-8

The original article [1] contained an error in Fig. 3B which was given wrong marking figures of NC.U87(0h) and KD.U87(0h).

**Fig. 3:**
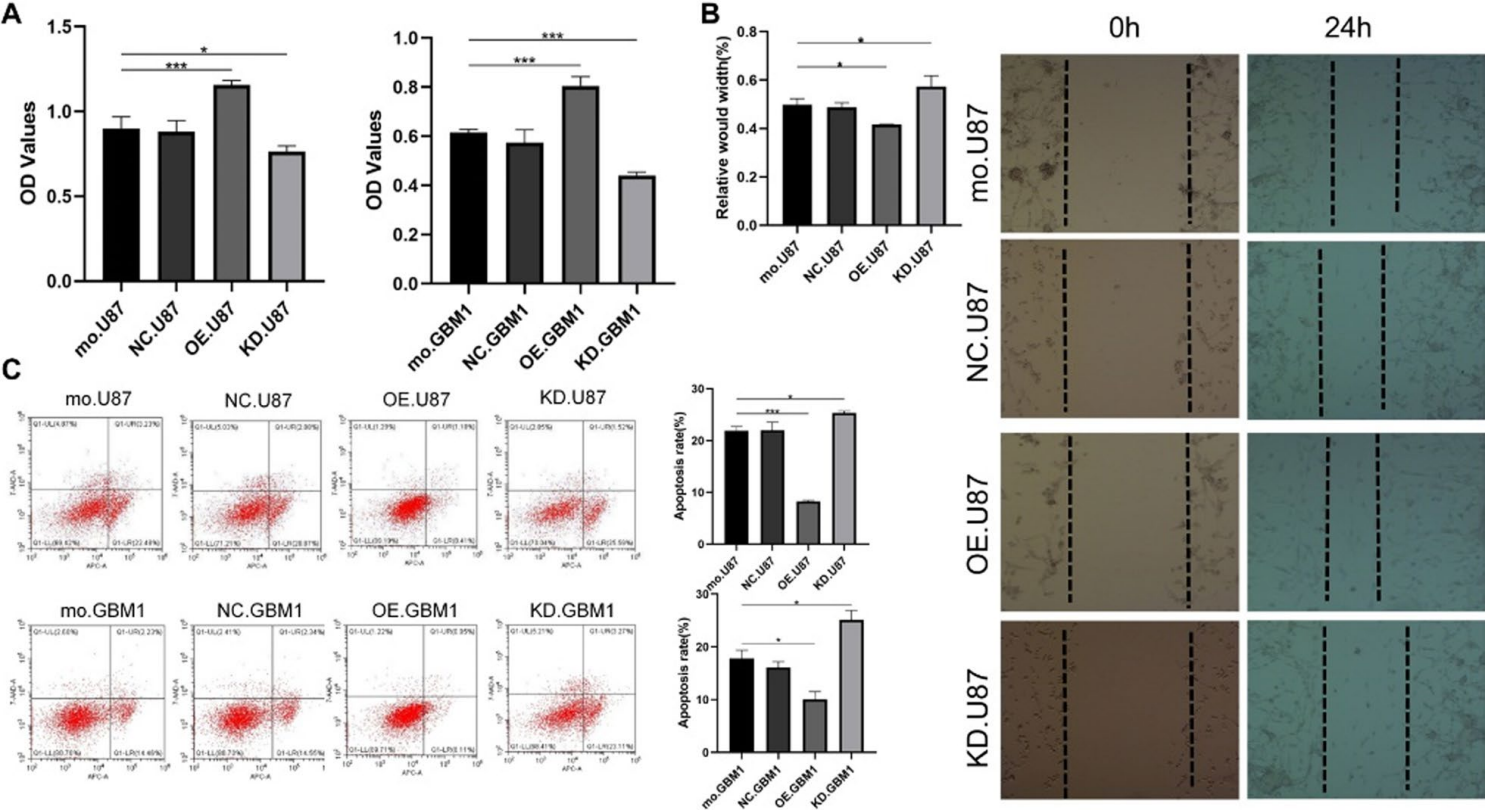
FOXS1 overexpression induces TMZ resistance in glioma cells. The proliferation (**A**), wound-healing (**B**) and apoptosis assay (**C**) of glioma cells with different FOXS1 expression under 200 μM TMZ treatment. control, normal control; NC, negative control; KD, knockdown; OE, overexpression (n ≥ 3, *P < 0.05, ***P < 0.001)

The original figures and data of Fig. 3 were checked by the authors and the editors of this article, no more figure or data was changed.

The corrected Fig. 3 is presented ahead.

## References

[1] Bing-zhou X (2021). CD90^low^ glioma-associated mesenchymal stromal/stem cells promote temozolomide resistance by activating FOXS1-mediated epithelial-mesenchymal transition in glioma cells. Stem Cell Res Ther.

