# Supplementary figures and images for: CD90low glioma-associated mesenchymal stromal/stem cells promote temozolomide resistance by activating FOXS1-mediated epithelial-mesenchymal transition in glioma cells
Source: Stem Cell Res Ther. 2021 Jul 13;12:394. doi: 10.1186/s13287-021-02458-8 (PMC8278613; doi:10.1186/s13287-021-02458-8)

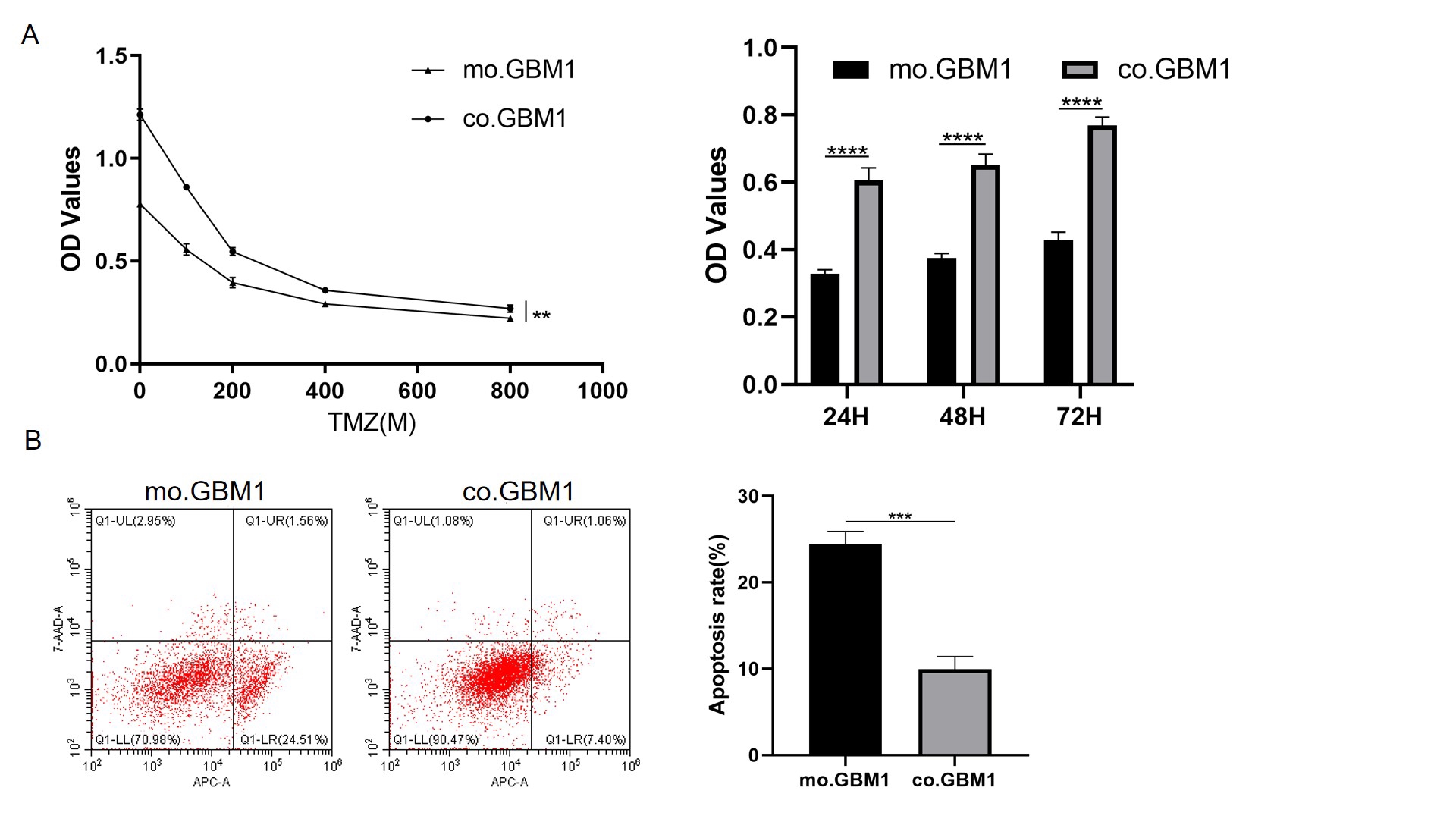

Supplement: Supplementary file 1 — Additional file 1: Supplementary Figure 1. Glioblastoma primary cell GBM-1 show increased TMZ resistance ability in gaMSCs-conditioned media in vitro. Proliferation curve (A) and apoptosis assay (B) of mo.GBM-1 and co. GBM-1 under TMZ treatment .(n ≥ 3,**P < 0.01,***P < 0.001,****P < 0.0001) [file 13287_2021_2458_MOESM1_ESM.jpg]
